# Transthyretin Protects against A-Beta Peptide Toxicity by Proteolytic Cleavage of the Peptide: A Mechanism Sensitive to the Kunitz Protease Inhibitor

**DOI:** 10.1371/journal.pone.0002899

**Published:** 2008-08-06

**Authors:** Rita Costa, Frederico Ferreira-da-Silva, Maria J. Saraiva, Isabel Cardoso

**Affiliations:** 1 Molecular Neurobiology, IBMC (Instituto de Biologia Molecular e Celular), Porto, Portugal; 2 Protein Production and Purification Unit, IBMC (Instituto de Biologia Molecular e Celular), Porto, Portugal; 3 ICBAS, University of Porto, Porto, Portugal; University of Arkansas for Medical Sciences, United States of America

## Abstract

Alzheimer's disease (AD) is a neurodegenerative disorder characterized by the deposition of amyloid β-peptide (A-Beta) in the brain. Transthyretin (TTR) is a tetrameric protein of about 55 kDa mainly produced in the liver and choroid plexus of the brain. The known physiological functions of TTR are the transport of thyroid hormone T_4_ and retinol, through binding to the retinol binding protein. TTR has also been established as a cryptic protease able to cleave ApoA-I *in vitro*. It has been described that TTR is involved in preventing A-Beta fibrilization, both by inhibiting and disrupting A-Beta fibrils, with consequent abrogation of toxicity. We further characterized the nature of the TTR/A-Beta interaction and found that TTR, both recombinant or isolated from human sera, was able to proteolytically process A-Beta, cleaving the peptide after aminoacid residues 1, 2, 3, 10, 13, 14,16, 19 and 27, as determined by mass spectrometry, and reversed phase chromatography followed by N-terminal sequencing. A-Beta peptides (1–14) and (15–42) showed lower amyloidogenic potential than the full length counterpart, as assessed by thioflavin binding assay and ultrastructural analysis by transmission electron microscopy. A-Beta cleavage by TTR was inhibited in the presence of an αAPP peptide containing the Kunitz Protease Inhibitor (KPI) domain but not in the presence of the secreted αAPP derived from the APP isoform 695 without the KPI domain. TTR was also able to degrade aggregated forms of A-Beta peptide. Our results confirmed TTR as a protective molecule in AD, and prompted A-Beta proteolysis by TTR as a protective mechanism in this disease. TTR may prove to be a useful therapeutic agent for preventing or retarding the cerebral amyloid plaque formation implicated in AD pathology.

## Introduction

Alzheimer's disease (AD), the most common form of dementia, is a brain disorder affecting the elderly and it is one of the causes of human disability and death in the developed world [1]. Pathologically, this disorder is characterized by intracellular neurofibrillary tangles, resulting from the accumulation of hyperphosphorylated microtubule-associated tau, as well as extracellular amyloid deposits called neuritic or senile plaques [2]; both lesions occur in the central nervous system, more specifically in the hippocampus and the cortex. These plaques are primarily composed of a peptide with 40–42/43 amino acids, amyloid beta peptide (A-Beta) which is formed through the sequential cleavage of the amyloid precursor protein (APP) by beta and gamma-secretases [3]. The accumulation of the peptide can be explained by an imbalance between its production and its degradation, although much less is known regarding this latest event [4]–[6].

The peptide and its precursor (APP) play important roles in neuronal functions; thus, control of physiological A-Beta levels, rather than complete inhibition seems to be an important strategy to reduce the accumulation of neuritic plaques and thus slowing down the progression of Alzheimer's disease [7]. Several molecules were identified and suggested as A-Beta carriers [8], [9]. Among them, transthyretin (TTR) has recently received a large attention [10]–[13]. TTR is a homotetrameric 55 kDa protein produced mainly in the liver and in the choroid plexus of the brain and secreted into plasma and cerebrospinal fluid (CSF), respectively [14]. Physiologically, it is responsible for thyroid hormone and retinol transport, through the binding of retinol binding protein [15]. More recently, a new function was attributed to TTR: experiments performed by Liz *et al* have demonstrated that TTR is a cryptic protease that cleaves Apolipoprotein A–I (Apo A–I) at its C-terminal [16], [17]. Over 100 TTR mutations have been associated with TTR related amyloid deposition in Familial Amyloidotic Polyneuropathy, affecting the peripheral nervous system.

Schwarzman and co-workers used CSF incubated with synthetic A-Beta (1–40) to identify A-Beta interacting proteins [8]. The authors concluded that TTR was the major A-Beta binding protein in CSF. A-Beta interaction with TTR resulted in a decrease in the aggregation state of the peptide as well as in its toxicity [8], [18]. A sequestration hypothesis was then considered: normally produced A-Beta is sequestered by certain extracellular proteins, thereby preventing amyloid formation and A-Beta cytotoxicity; formation of amyloid and the consequent toxicity occurs when sequestration fails [18]. This interaction was further characterized by our group and the respective Kd was determined as 28±5 nM. WT TTR was observed to interact with all the A-Beta forms tested (soluble, oligomers and fibrils), with similar strengths and was able to inhibit the aggregation and disrupt preformed A-Beta fibrils, behaving as well as a protective molecule in cell culture through inhibition of apoptosis [19]. *In vivo* experiments were performed to investigate the protective effect of TTR: in *Caenorhabditis elegans* expressing human A-Beta (1–42), TTR rescued the neurodegeneration triggered by the toxic peptide [20]; studies in transgenic mice overexpressing mutant APP revealed slower disease progression and lack of neurodegeneration attributed to the increased expression of several neuroprotective genes, including TTR [11]; in the absence of TTR, A-Beta deposition was accelerated in transgenic mice models [15], [21].

The discussion on TTR/A-Beta interaction and consequent inhibition of aggregation and toxicity reduction raised the hypothesis that mutations in the TTR gene or conformational changes in the protein induced by aging, could affect the sequestration properties. However, no mutations in the TTR gene have been found in AD patients [22]. Recent work investigated binding of A-Beta to several TTR variants; TTR variants with high amyloidogenic potential bound poorly, whereas more stable variants had a higher affinity for A-Beta [19].

The total A-Beta burden is a balance between production and accumulation; besides sequestration, clearance is also achieved by catabolic processing of the peptide. Proteases, acting in multiple cellular compartments, are important to degrade the peptide [7]. These include Neprilysin (NEP), Insulin degrading enzyme (IDE), Endothelin-converting enzyme (ECE), angiotensin-converting enzyme (ACE), uPA/tPA-plasmin system, cathepsin D, and matrix metalloendopeptidase 9 [4], [23]. NEP and IDE are metalloproteases widely expressed in many normal tissues; in the brain they are localized in areas vulnerable to amyloid deposition. NEP is able to cleave not only monomeric but also oligomeric A-Beta forms both localized intracellularly and extracellularly. The role of these enzymes in A-Beta degradation was confirmed by *in vitro* and *in vivo* studies [7], [24], [25]. These studies confirmed diminished A-Beta deposition, when enzymes were overexpressed; levels of mRNA and expressed enzymes significantly decreased in AD brains, specifically in regions most vulnerable to AD, as compared with healthy controls [24].

In this work, we investigated mechanisms underlying TTR protection against A-Beta aggregation and toxicity, and showed that A-Beta is processed proteolytically by TTR, resulting in smaller less amyloidogenic peptides.

## Materials and Methods

### A-Beta peptides

A-Beta peptides (1–42) and (1–40) from BioSource and A-Beta peptides (1–14) and (15–42) synthesized at Rpeptide were dissolved in hexafluorisopropanol (HFIP) and kept at room temperature for 1–2 hours. The HFIP was then removed under a stream of nitrogen until a clear film remained in the microcentrifuge tube. The residue was then dissolved in DMSO at 2 mM.

### TTR production and purification

Human recombinant TTRs were produced in a bacterial expression system using *Escherichia coli* BL21 [26] and purified as previously described [27]. Briefly, after growing the bacteria, the protein was isolated and purified by preparative gel electrophoresis after ion-exchange chromatography. The same protocol was used to isolate TTR from human serum: serum was dialysed against 50 mM phosphate buffer and 77 mM NaCl (pH 7.6) and chromatographed on an ion-exchange DEAE-cellulose column. The protein was eluted by increasing ion strength. The TTR fraction was then re-chromatographed in a gel-filtration column. Protein concentration was determined using the Lowry method [28].

### A-Beta cleavage products by TTR analysed by SDS-PAGE and imunoblot

Human, recombinant or sera, TTR (15 µg) and A-Beta peptide (1–42) (2 µg) were incubated in 20 µl 50 mM Tris, pH 7.5, at 37°C for different periods of time, and then reactions were applied onto a 15% SDS-PAGE gel and visualized by coomassie blue staining. Alternatively, TTR incubated with A-Beta (1–42) and A-Beta alone, were run on a SDS-PAGE gel, transferred to nitrocellulose membranes, and A-Beta immunodetected using an anti-A-Beta antibody (BAM-10, Sigma). In another set of experiments, A-Beta was incubated for 6 hours at 37°C for aggregation purposes and then subsequently incubated with TTR. Results were visualized by immunoblot, as described above.

To evaluate possible inhibitors of A-Beta proteolysis by TTR, recombinant TTR (15 µg) was pre-incubated for 30 minutes at 37°C, either with 1 mM pefabloc, 0.33 µM αAPP of peptide encompassing aminoacids 18–688, containing the KPI domain (Neuromics) or with 0.33 µM αAPP peptide derived from the APP isoform 695 (without the KPI domain) (Sigma), both formed by a α-secretase cleavage. Then, A-Beta was added (2 µg) in a final volume of 20 µl, and further incubated for 6 hours at 37°C. Results were observed by immunoblot as described. Experiments were repeated at least twice.

### Analysis of A-Beta cleavage products

#### Mass spectrometry

For analysis of A-Beta cleavage products, a fresh mixture of TTR (30 µg) and A-Beta (4 µg) reaction was submitted to molecular weight determination by MALDI-TOF/TOF mass spectrometry (Applied Biosystems 4700 Proteomics Analyzer). For control experiments, albumin (Sigma) or gelsolin (Sigma) was incubated with A-Beta peptide and analyzed as above. To determine the amino sequence of newly observed peaks, MS/MS peptide de novo sequencing using a specific software program (Applied Biosystems DeNovo Explorer) was performed.

#### Reversed Phase chromatography

A-Beta (40 µg), recombinant TTR (192 µg) and mixtures of the two were incubated in 50 mM Tris, pH 7.5, at 37°C, for different periods of time. The reactions were stopped by diluting mixtures with 1% aqueous trifluoroacetic acid (TFA) to obtain a final A-Beta concentration of 19 µM. A-Beta and fragments were resolved by reversed-phase HPLC (RP-HPLC) using an AKTÄ purifier 10 (GE Healthcare Bio-Sciences AB) with a LichroCart 250-4 Lichospher 100 RP-18 (Merck) pre-equilibrated in buffer A (0.1% TFA in water). The elution occurred with a 0–60% B (0.1%TFA in acetonitrile) gradient over 10 column volumes. The flow rate was 1 ml/min and elution was monitored by absorbance at 206 nm. Half minute fractions were collected (Frac-920, GE Healthcare Bio-Sciences AB) and peaks were analyzed by mass spectrometry and N-terminal sequencing.

#### Thioflavin T (Th T) binding assay

100 µg of each peptide were diluted in F12 media at 100 µM and incubated at 37°C for different periods of time. Excitation spectra were recorded on a Jasco FP-770 spectrofluorometer at 25°C with 30 µM Th T (Fluka) in 50 mM glycine/NaOH buffer, pH 9.0 in a 1-ml assay volume. Results were presented by plotting the intensity of fluorescence at 450 nm which is the characteristic novel excitation maxima formed upon Th T binding. Experiments were repeated at least twice.

#### Transmission Electon Microscopy (TEM)

A-Beta (1–14), (15–42) and (1–42) peptides from a 2 mM stock were diluted to 100 µM in F12 media (Gibco BRL) and incubated at 37°C for 5 days; to analyze the action of the αAPP peptide containing the KPI domain (Neuromics) and the αAPP peptide derived without the KPI domain on the inhibitory effect of TTR on A-Beta aggregation, each peptide (0.33 µM) was pre-incubated with TTR (10 µM) for 30 min; then A-Beta (100 µM) was added and the mixture further incubated for 15 hours at 37°C.

For visualization by TEM, sample aliquots were absorbed to glow-discharged, carbon-coated collodion film supported on 200-mesh copper grids, and negatively stained with 1% uranyl acetate. The grids were exhaustively visualized with a Zeiss microscope (model EM10C), operated at 60 kV. Experiments were repeated at least twice.

#### Statistical analyses

All data examined were expressed as mean±S.E. Comparison between groups was made using the Student's t test. A p value of less than 0.05 was considered statistically significant.

## Results

### TTR cleaves A-Beta

**Figure 1:**
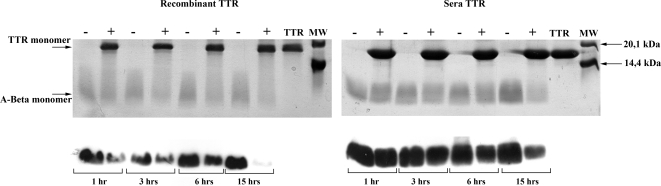
Kinetics of A-Beta proteolysis by TTR. 2 µg of A-Beta were incubated alone (−) or in the presence of 15 µg of human TTR (+), either isolated from sera (right panels) or recombinant (left panels), for different periods of time: 1 hour (1 hr), 3 hours (3 hrs), 6 hours (6 hrs) and 15 hours(15 hrs), at 37°C. Visualization was performed either by staining the SDS-PAGE gel with Coomassie Blue (upper panels) or by western blot of the gel using an antibody specific for A-Beta (lower panels). Bands corresponding to the TTR and A-Beta (1–42) monomers are indicated by arrows (left side) as well as the molecular weight standards used (right side); TTR incubated for 15 hrs at 37°C was also run on the gel (TTR); MW- molecular weight standards.

We have previously shown that TTR binds A-Beta, inhibits its aggregation and consequently abrogates its toxicity [19]. To characterize the nature of the protective role of TTR on A-Beta toxicity and since TTR has proteolytic activity [16], we hypothesized that TTR could cleave A-Beta and thus, contribute to its clearance. By SDS-PAGE analysis, we observed that the band corresponding to the A-Beta (1–42) monomer was less intense in preparations of recombinant TTR incubated with A-Beta (1–42) as compared with A-Beta incubated alone (Figure 1). Similar experiments carried out with A-Beta (1–40) peptide resulted in analogous observations (not shown). This prompted our attention to the possibility of proteolytic phenomena. We could not detect new bands in the gel, corresponding to putative generated fragments, which can be explained by the small size of cleavage products.

Kinetics studies showed that this effect was evident as early as 1 hour of co-incubation, increasing with time (Figure 1, top left panel). To verify the identity of the weaker band, we performed western blot followed by immunodetection using an A-Beta specific antibody (Figure 1, bottom left panel) and we confirmed that the weaker band was immunoreactive. At this point we also assayed different TTR batches to further validate the ability of TTR at cleaving the A-Beta peptide, excluding the possibility of being a feature of a single TTR batch, or of contaminants present in a particular preparation; in all cases, co-incubation of TTR with A-Beta resulted in a weaker A-Beta monomer band, when compared with A-Beta incubated alone (not shown).

To further explore this hypothesis and exclude the possibility of contaminants associated to the protocol of recombinant protein purification from bacteria, incubation of A-Beta (1–42) with human TTR isolated from serum was performed. Results are displayed in Figure 1 and support the above results, since the amount of A-Beta in the preparation incubated with TTR is lower than in the preparation of A-Beta alone. The kinetics followed the trend observed with recombinant TTR either detected by coomassie blue staining (Figure 1, top right panel) or by immunodetection (Figure 1, bottom right panel); TTR from different donors produced the same effect (not shown).

### Identification of the cleavage site on A-Beta peptide

**Figure 2:**
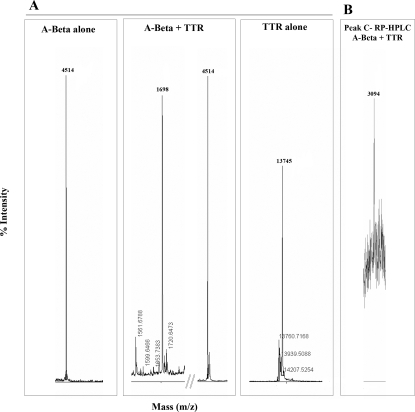
A-Beta proteolysis by TTR analyzed by mass spectrometry. A- Preparations of A-Beta alone, A-Beta incubated with TTR (A-Beta+TTR), or TTR alone incubated for 3 hours at 37°C, analyzed by MALDI-TOF/TOF mass spectrometry. The new 1698 Da peak present in A-Beta+TTR preparations was submitted to MS/MS peptide de novo sequencing, and showed to correspond to the first 14 aminoacid residues of A-Beta peptide. B- Fraction corresponding to peak c of A-Beta incubated with TTR for 15 hours at 37°C, and subjected to RP-HPLC, and also analyzed by mass spectrometry, showed a peak of approximately 3094.7 Da which indicates the presence of A-Beta peptide 1–27.

To further characterize A-Beta proteolytic processing by TTR, and establish the cleavage site or sites in A-Beta (1–42), we started by determining the molecular weight of proteins/peptides present in preparations after 3 hours incubation, using both recombinant and serum TTR. We observed a new peak of 1698 Da in the TTR/A-Beta (1–42) mixture whereas in the separate A-Beta (1–42) and TTR preparations only the expected 4517 Da and 13745 Da peaks were observed, respectively (Figure 2A). Control experiments were performed using preparations of A-Beta co-incubated with albumin which did not reveal any new peak (not shown). Gelsolin, a known A-Beta ligand was also assessed and failed to generate new A-Beta peaks (not shown). The aminoacid sequence of the new peptide was determined by *de novo* sequencing of the 1698 Da peak which revealed the first 14 aminoacids of the A-Beta peptide sequence. Analysis of 15 hours incubations revealed the 1698 Da peak and in addition peaks of 1010, 1562 and 1955 Da (not shown). Although we could not perform *de novo* sequencing on these three peaks, they probably correspond to aminoacids 3–10, 1–13 and 1–16, respectively, accordingly to the results obtained using the Isotopident program (http://education.expasy.org/student_projects/isotopident/htdocs/).

### Amyloidogenecity of new A-Beta peptides

**Figure 3:**
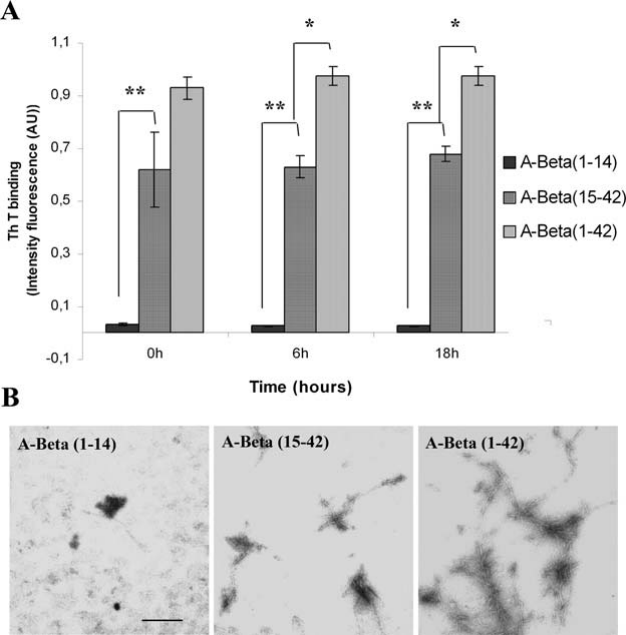
Amyloidogenic potential of A-Beta peptides (1–14) and (15–42). A- Th T binding assay of A-Beta peptides; A-Beta (1–14) does not bind Th T whereas peptide (15–42) presents amyloidogenic potential which is lower than the (1–42) counterpart. Results were presented by plotting the intensity of fluorescence at 450 nm which is the characteristic novel excitation maxima formed upon Th T binding for each peptide; AU-arbitrary units. * p<0.02; ** p<0.0006. B- Ultrastructural analysis by TEM of A-Beta peptides incubate for 5 days at 37°C. Peptide (1–14) did not form amyloid fibrils; peptide (15–42) formed fibrils under these circumstances but shorter and less abundant than A-Beta (1–42). Scale bar = 500 nm.

We next evaluated amyloidogenecity of the smaller generated peptides as compared to A-Beta (1–42), by Th T binding studies and by TEM ultrastructural analysis. The fragment (1–14) failed to bind Th T and to produce the new characteristic peak at 450 nm, even after 24 hours at 37°C (Figure 3A). A-Beta (15–42) showed aggregation properties as significant binding to Th T was observed, although less intense than that of full-length peptide.

Ultrastructural studies depicted in Figure 3B corroborated the Th T assays since no fibrils were detected by TEM in the A-Beta (1–14) preparation; A-Beta (15–42) retained the ability to aggregate, but fibrils were shorter and less abundant than in the preparation of A-Beta (1–42). Taken together, these results showed that A-Beta amyloidogenecity is diminished upon cleavage by TTR.

### Reversed phase chromatography

**Figure 4:**
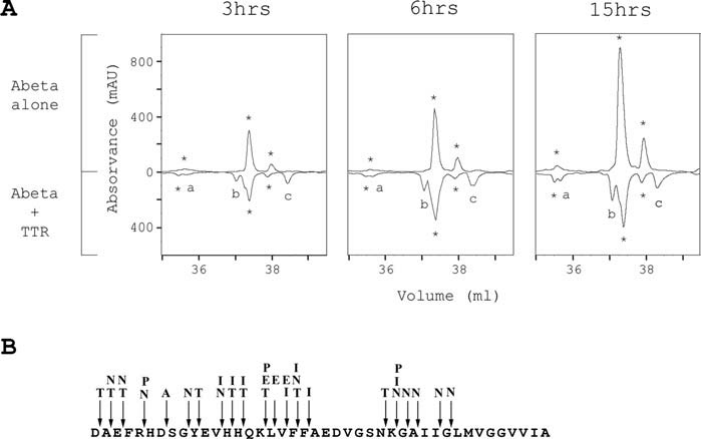
A-Beta peptide cleavage by TTR. A- RP-HPLC profiles of A-Beta proteolysis by TTR. 40 µg A-Beta was incubated with TTR (192 µg) for different periods of time (3, 6, and 15 hours). Enzymatic hydrolysis was stopped and samples were subjected to RP-HPLC analysis as described in the Material and Methods section. B- A-Beta (1–42) aminoacid sequence and the cleavage sites of A-Beta by various enzymes including NEP (N), IDE (I), ACE (A), ECE (E), plasmin (P) [25] and TTR (T).

In an attempt to identify additional A-Beta cleavage sites corresponding to fragments failing to ionize, we analyzed, by reversed phase chromatography, A-Beta and A-Beta/TTR preparations incubated for different periods of time. Figure 4 shows the chromatographic profiles obtained after 3, 6 and 15 hours. After 3 hours incubation, 3 peaks were identified: peaks a, b and c (Figure 4A, lower chromatograms), as compared with the A-Beta alone profile (Figure 4A, upper chromatograms); the intensities of these peaks increased over-time. Peaks marked with an asterisk, also present in preparations corresponding to A-Beta alone, increased with time, probably due to aggregation of A-Beta; for the same time-point these peaks decreased with TTR.

N-terminal sequence performed for 5–6 cycles in fractions corresponding to peaks a, b and c, revealed A-Beta cleaved between aminoacids 19–20 in peak a; A-Beta cleaved between aminoacids 1–2 and 3–4 in peak b; peak c revealed the first 5 aminoacids of the A-Beta peptide sequence. Peak c could represent a new A-Beta aggregation state or a peptide cleaved after position 5. Mass spectrometry analysis of this fraction showed a peak of 3094 Da (Figure 2B), suggesting another cleaving site between aminoacids 27–28. Figure 4B shows the A-Beta (1–42) aminoacid sequence and the cleavage sites of A-Beta by various enzymes including NEP (N), IDE (I), ACE (A), ECE (E), plasmin (P), as previously described [25], and TTR (T) as determined in this work.

### APP peptides with the KPI domain inhibit A-Beta (1–42) cleavage by TTR

**Figure 5:**
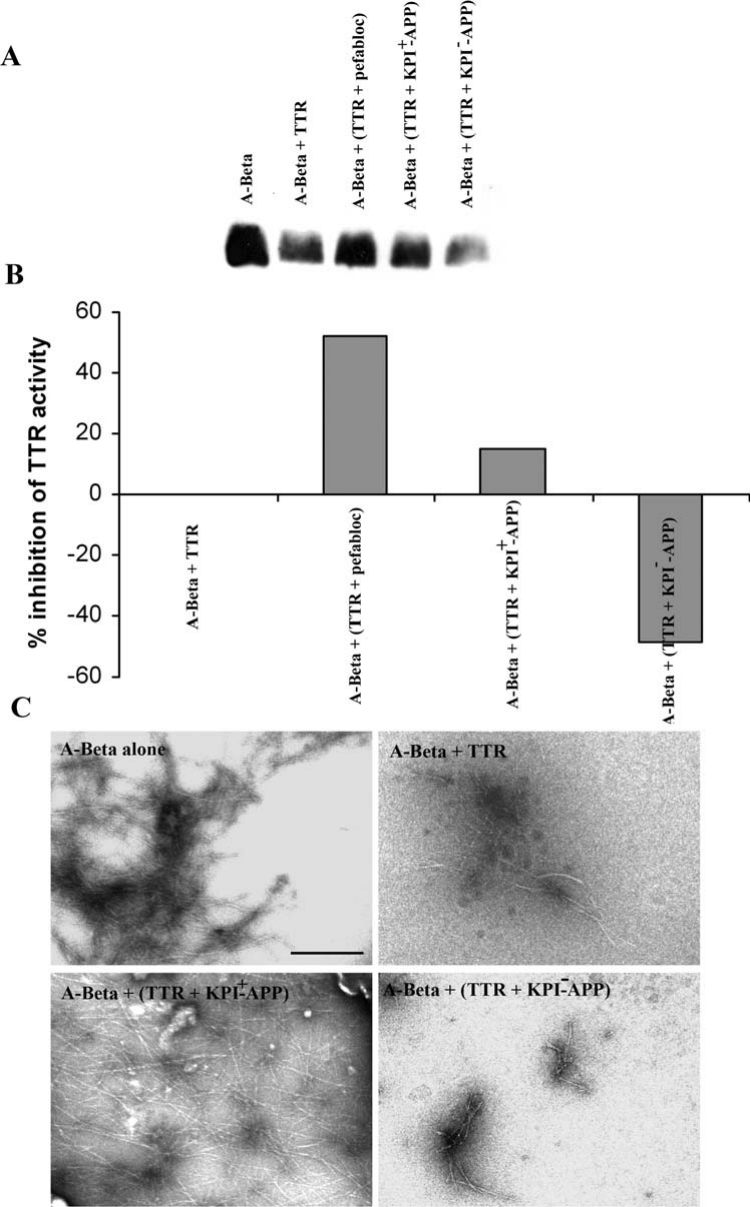
A-Beta proteolysis by TTR is KPI-sensitive. A- A-Beta incubated with TTR (A-Beta+TTR) shows a weaker A-Beta monomer band as compared to A-Beta alone (A-Beta), indicative of proteolysis, as analyzed by SDS-PAGE electrophoresis followed by western blot. Pre-incubation of TTR with pefabloc (A-Beta+(TTR+pefabloc)) and with an αAPP peptide containing the KPI domain (A-Beta+(TTR+KPI^+^−APP)) inhibits TTR proteolytic activity, whereas the αAPP peptide without the KPI domain (A-Beta+(TTR+KPI^−^−APP)) facilitates proteolysis. B- % of inhibition of TTR proteolysis by quantification of band intensity in A. C- Ultrastructural analysis by TEM of preparations incubated for 15 hours, as described in Materials and Methods. TTR inhibited A-Beta aggregation as compared with A-Beta incubated alone (upper panels). Pre-incubation of TTR with αAPP peptide containing the KPI domain (A-Beta+(TTR+KPI^+^−APP)) abrogated TTR ability to avoid A-Beta aggregation, whereas αAPP lacking the KPI domain (A-Beta+(TTR+KPI^−^−APP)) did not affected TTR activity (lower panels). Scale bar = 500 nm.

TTR is a cryptic protease able to cleave Apo A-1 and this cleavage is inhibited by PMSF, Pefabloc, and others [16] and thus we tested if A-Beta proteolytic processing by TTR was also inhibited by the same molecules. Analysis of A-Beta after incubation with TTR in the presence or absence of Pefabloc was investigated by immunoblot (Figure 5A). Densitometry analysis indicated an inhibition of TTR proteolytic activity of approximately 52% by pefabloc (Figure 5B). APP isoforms with the KPI domain are associated with increased A-Beta deposition [29]. To gain insights into the proteolytic mechanism by which TTR cleaves and protects against A-Beta toxicity, we tested the ability of 2 APP peptides formed by α-secretase cleavage to block A-Beta (1–42) cleavage by TTR. Analysis of the results was performed by SDS-PAGE, followed by immunodetection, and is shown in figure 5A. One of the peptides derived from the 770 isoform and containing the KPI domain partially abolished proteolysis of A-Beta by TTR, by approximately 15% (Figure 5B). The other used peptide, derived from the APP isoform 695 without KPI domain, did not inhibit A-Beta proteolysis by TTR; on the contrary, it seemed to favor cleavage of the peptide (Figures 5A and B). Structural analysis by TEM, depicted in Figure 5C, showed that the previously described inhibition of A-Beta aggregation by TTR [19] is abrogated by the presence of KPI-containing peptide but not by the KPI-lacking peptide, thus establishing proteolysis as the mechanism underlying inhibition of A-Beta fibrillization by TTR.

### TTR cleaves aggregated forms of A-Beta

**Figure 6:**
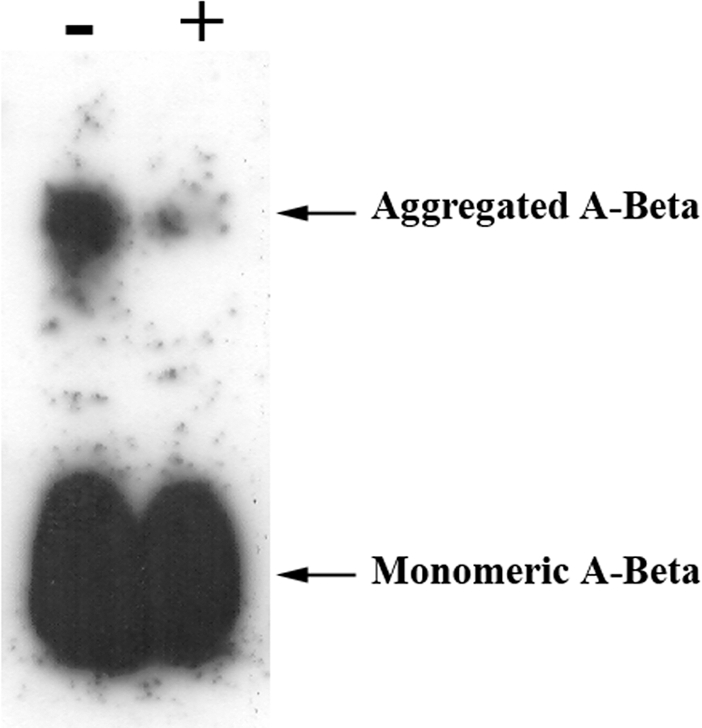
Influence of TTR in A-Beta aggregates. A-Beta alone was incubated for 3 hours at 37°C to form aggregates and then further incubated with or without TTR for another 3 hours. Analysis was done by western blot following separation under denaturing conditions. Results indicated that in preparations of A-Beta incubated with TTR, both bands corresponding to A-Beta monomer and to the higher molecular form presented decreased intensities, as compared to the same bands in the A-Beta alone preparation.

We have previously shown that TTR binds to different A-Beta species namely soluble, oligomeric and fibrillar A-Beta. Furthermore, besides inhibiting its aggregation, TTR is also capable of disaggregating A-Beta mature fibrils [19]. In this line of thought, we next evaluated the possibility of TTR cleaving other forms of A-Beta and for that we analyzed the preparations by western blot. In this case, previously to the incubation with TTR, A-Beta peptide was incubated alone for aggregation purposes, and then further incubated in the presence or absence of TTR for another 3 hours. Results were visualized by western blot after separation under denaturing conditions, displayed in Figure 6 and showed that A-Beta higher molecular forms were also diminished in the samples containing TTR, implying that TTR also degrades aggregated forms of A-Beta.

## Discussion

TTR has been suggested as an A-Beta carrier [8]–[10], [18] and attempts to relate TTR levels and Alzheimer's disease have been made [30], [31]. Very recently, proteolytic activity has been attributed to TTR as a novel physiological function [16]. In this work, authors showed that TTR has a chymotrypsin-like serine protease activity, capable of cleaving apoA-1 *in vitro*. No other substrates were identified yet. Proteolysis has been viewed as an important event in most types of amyloidosis. On one side, in some cases, the amyloidogenic proteins/peptides are released from precursors by proteolysis. Examples include the A-Beta peptide [32], [33], the 34-residue Abri peptide [34], [35], the 50-residue medin peptide [36] and others [37]. On the other side, proteolysis can function to protect, providing a way for clearance of the amyloidogenic proteins/peptides. It is the case of A-Beta peptide degradation by several enzymes that participate in A-Beta catabolism, which include neprilysin, insulin-degrading enzyme (IDE), endothelin-converting enzyme (ECE) and angiotensin-converting enzyme (ACE), metalloproteinase-9 (MMP-9) and plasmin [25]. Many of them have more than one cleavage site in A-Beta [25], (see figure 4B).

Here we showed that TTR also cleaves A-Beta (1–42) and several cleavage sites were identified. Proteolysis occurred after bulky hydrophobic amino acid residues as tyrosine, and phenylalanine; after positively-charged amino acid residues as lysine; after small neutral amino acid residues as alanine; but also after other aminoacid residues as aspartic acid, glutamic acid, histidine and asparagine, which are not part of the common aminoacid residues used by serine proteases. Nevertheless, cleavage after the negatively charged glutamic acid and after an aspartic acid has been described for serine proteases [38]: Staphylococcal exfoliative toxins A and B cleave the peptide β-melanocyte-stimulating hormone after specific glutamic acid residues; the crystal structure of the toxins also indicated that the enzyme probably also cleaves after an aspartic acid, and other residues as histidines and asparagines were also identified. Several of these TTR cleaving sites are also sites for other A-Beta degrading enzymes, suggesting these are aminoacid residues in A-Beta prone to proteolysis. A-Beta degradation by TTR, together with the action of other A-Beta degrading enzymes, will contribute to its clearance; the newly generated peptides can be eliminated by the cells as they are not toxic or not as toxic as the 1–42 peptide precursor. Since A-Beta (15–42) can be further cleaved by TTR in smaller peptides, it is expected to lose its partial amyloidogenic potential. This finding together with the demonstrated protection of TTR against A-Beta toxicity, as assessed by caspase-3 activation, previously described [19], is suggestive of proteolysis as one possible mechanism of TTR protection in AD.

It has been reported that A-Beta generated in the brain can be bound to cholesterol/apoE and in this way cross the blood brain barrier (BBB), to be further incorporated in HDL for delivery in the liver [39]; thus, it was important to assess A-Beta cleavage by TTR in sera; our results demonstrated that isolated sera TTR was able to cleave A-Beta peptide (1–42), therefore indicating that A-Beta can be cleaved by TTR also in the liver and/or sera.

Our results indicated that TTR has also a proteolytic effect on aggregated A-Beta as analyzed by western blot. This, together with previous findings showing that TTR was able to bind and disrupt A-Beta fibrils [19], suggests that, *in vivo*, TTR can also develop its proteolytic activity on A-Beta deposits. TTR has been localized within senile plaques [12], [40] in AD, either in clinical samples and animal models. In this way, TTR contributes not only to the maintenance of A-Beta levels within normal range but also to the removal of deposited A-Beta in case of imbalance and disease, as it seems to be the case of other A-Beta degrading enzymes such as neprilysin [24].

APP is presented at least in three isoforms resulting from alternative splicing [41]. The isoform 695 is predominantly generated in neuronal cells whereas isoforms 751 and 770 are more abundant in astroglial cells and contain a KPI domain [42]. Curiously, these two isoforms are associated with increased A-Beta deposition [29] and the respective mRNAs are increased in AD brains [41]. It has been already suggested that this may be related to the inhibitory effect of KPI on serine proteases [43]. In fact, other reports describe that KPI-containing APP isoforms regulate extracellular cleavage of secreted αAPP [41], resulting in increased A-Beta (1–42) production [44]. Here, we showed that TTR ability to cleave A-Beta peptide (1–42) was inhibited in the presence of an αAPP secreted form (aminoacids 18–688) containing the referred domain, supporting the idea of a TTR protective role via proteolysis. Unexpectedly, the αAPP peptide derived from an isoform lacking the KPI domain, not only did not inhibit TTR proteolytic activity but on the contrary, facilitated the cleavage. Secreted αAPP has been described as neuroprotector, both *in vivo* and *in vitro* [12] and in this context the KPI-containing isoform should also provide for protection. However, a careful analysis of the literature indicates that in most studies, including AD mouse models, the APP isoform used was the 695 one, thus lacking the KPI domain. Therefore, one can hypothesize that αAPP forms which are neuroprotective derive solely from the APP isoforms without the KPI domain, and that the mechanism underlying neuroprotection involves increasing A-Beta degradation and clearance. Other reports describe that genetic reduction of TTR increases A-Beta steady-state levels in the brain, both soluble and detergent extractable, but does not alter APP processing [13]. Therefore, A-Beta peptide elevation levels found in this model can result from deficient clearance due to decreased proteolysis by TTR.

In summary, here we showed for the first time that TTR cleaves A-Beta and identified several cleavage sites. At this point we cannot rule out the possibility of other cleaving sites. TTR also inhibits A-Beta fibrillogenesis and toxicity and thus, we hypothesized that A-Beta proteolysis by TTR contributes to its clearance as the amyloidogenic potential of the new peptide generated is null or lower than that of the full-length peptide. We also demonstrated that the referred cleavage is abolished by an APP peptide containing the KPI domain. This result can explain, at least partially, the increased A-Beta deposition in sites related to APP isoforms containing the KPI domain, since TTR proteolytic activity is probably inhibited under these circumstances. Cells with abundant APP isoforms lacking the KPI domain, facilitate TTR activity and thus, have less or no A-Beta deposition.

Targeting A-Beta clearance represents a potential therapeutic avenue for patients with AD; A-Beta degrading proteases-based therapies represent an alternative approach in controlling A-Beta clearance. It is known that TTR is decreased in the CSF of AD patients [30] and thus TTR may prove to be a useful therapeutic agent for preventing or retarding the cerebral amyloid plaque formation implicated in AD pathology.
